# Supplementary material for: APC/CCDH1 synchronizes ribose-5-phosphate levels and DNA synthesis to cell cycle progression
Source: Nat Commun. 2019 Jun 7;10:2502. doi: 10.1038/s41467-019-10375-x (PMC6555833; doi:10.1038/s41467-019-10375-x)
Supplement: Supplementary file 1 — Supplementary Information [file 41467_2019_10375_MOESM1_ESM.pdf]

APC/C^{CDH1} Synchronizes Ribose-5-Phosphate Levels and DNA Synthesis to Cell Cycle Progression

Li et al.

1. **Supplementary Figures 1-10**
2. **Supplementary Table 1**

a

HeLa cells released after Double thymidine synchronization

Hours	0	1	2	3	4	5	6	7	8	9	10	11
G_1(%)	96.54	88.86	15.21	15.04	6.5	6.71	6.78	11.62	31.95	59.49	79.07	84.96
S(%)	4.49	8.26	87.53	87	19.96	16.34	13.28	13.19	10.51	9.02	9.69	9.54
G_2/M(%)	0.02	0.01	2.66	3.73	71.71	77.83	79.62	76.35	53.98	30.76	13.74	1.36

b

HeLa cells released after RO3306 synchronization

Hours	0	0.5	1	2	4	8
G_1(%)	5	18.88	27.64	31.43	37.73	69.26
S(%)	24.28	23.28	30.15	18.1	15.42	13.64
G_2/M(%)	70.11	53.05	46.61	46.18	41.41	14.44

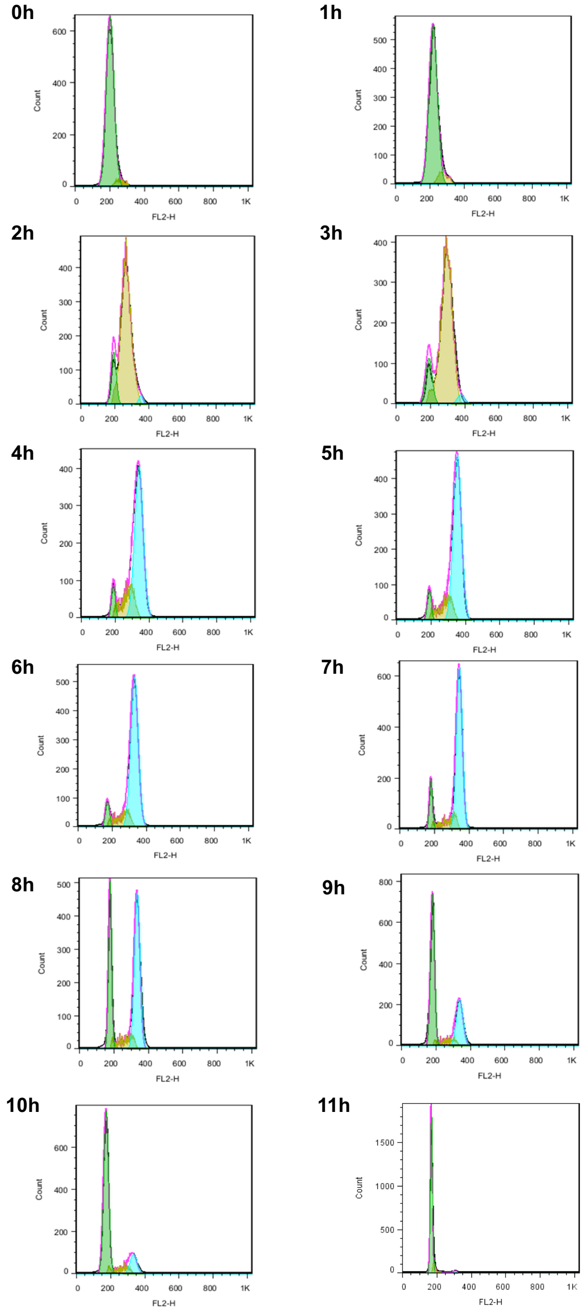

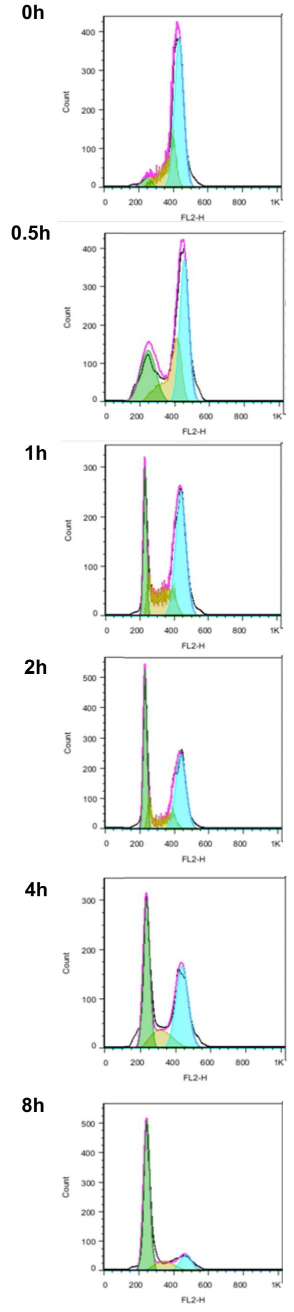

Supplementary Figure 1. Cell phases after release (related to Fig. 1). HeLa cell synchronized by double thymidine block (**a**) or RO3306 block (**b**) were released. Percentage (upper) of cells at different phases was determined by flow cytometric analysis (lower) at times indicated. The gating strategy of flow cytometry was shown in Supplementary Figure 9.

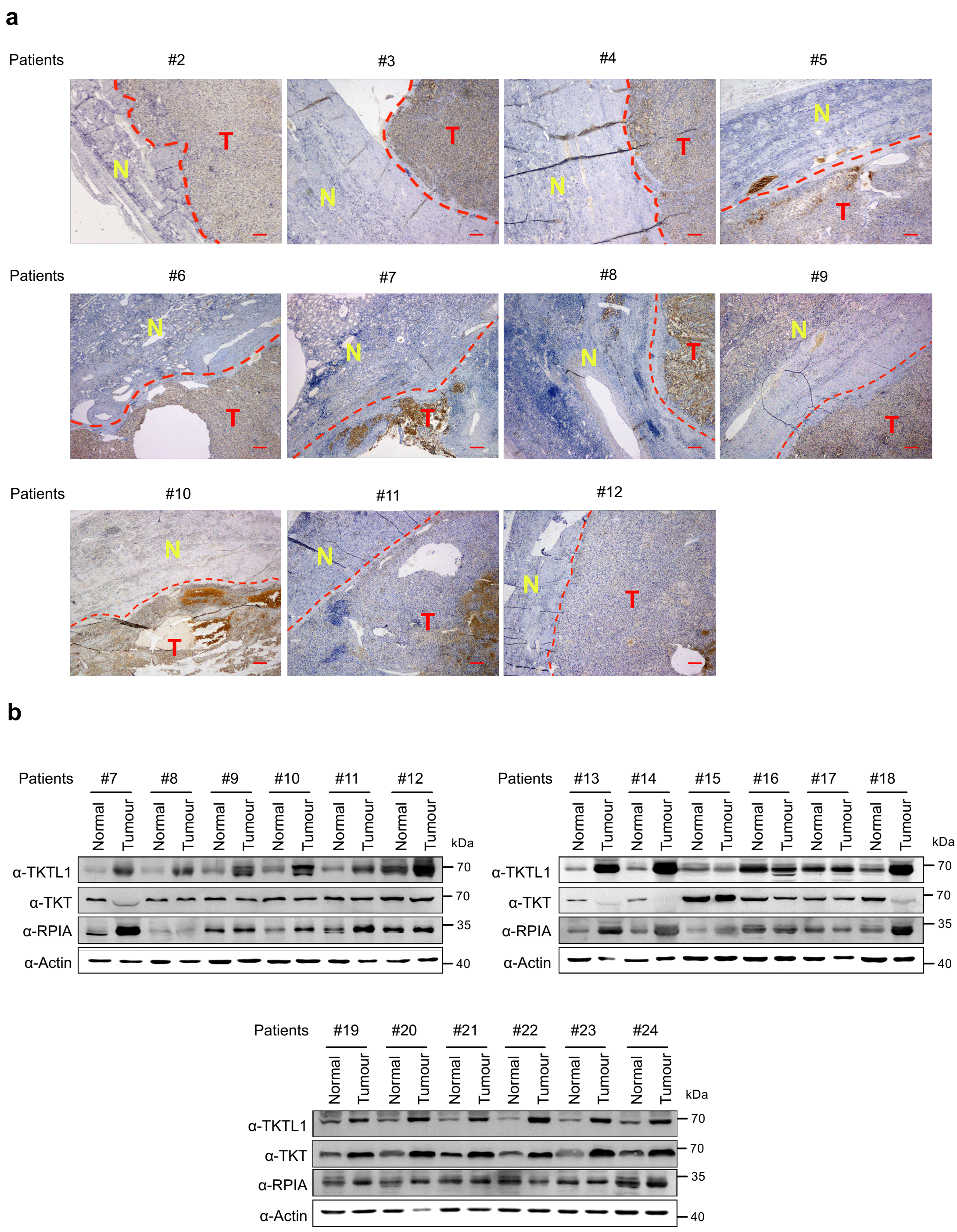

Supplementary Figure 2. Overexpression TKTL1 in ccRCC tissues (related to Fig. 1). (**a**) Immunohistochemically staining analysis of ccRCC and their adjacent non-cancer tissues. T: ccRCC tumor tissue; N: adjacent normal tissue. Scale bars: 200 μm. Quantitating results are presented in Fig. 1g. (**b**) Western blotting analysis for protein levels in ccRCC and matched adjacent normal tissues. Quantitating results are presented in Fig. 1h.

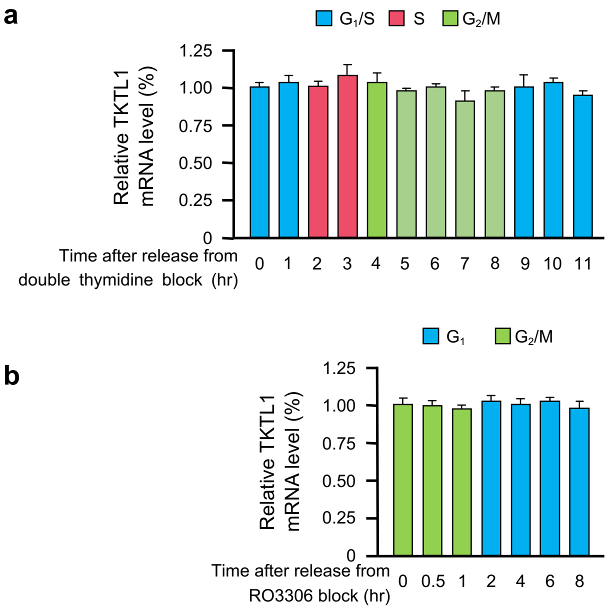

Supplementary Figure 3. TKTL1 is not regulated at transcription level during cell cycle (related to Fig. 2). *TKTL1* mRNA levels of HeLa cells during cell cycle progression released from in double thymidine synchronization (**a**) and RO3306 synchronization (**b**) were determined by qRT-PCR. Each column represents the mean ± SEM (n=5 biologically independent samples).

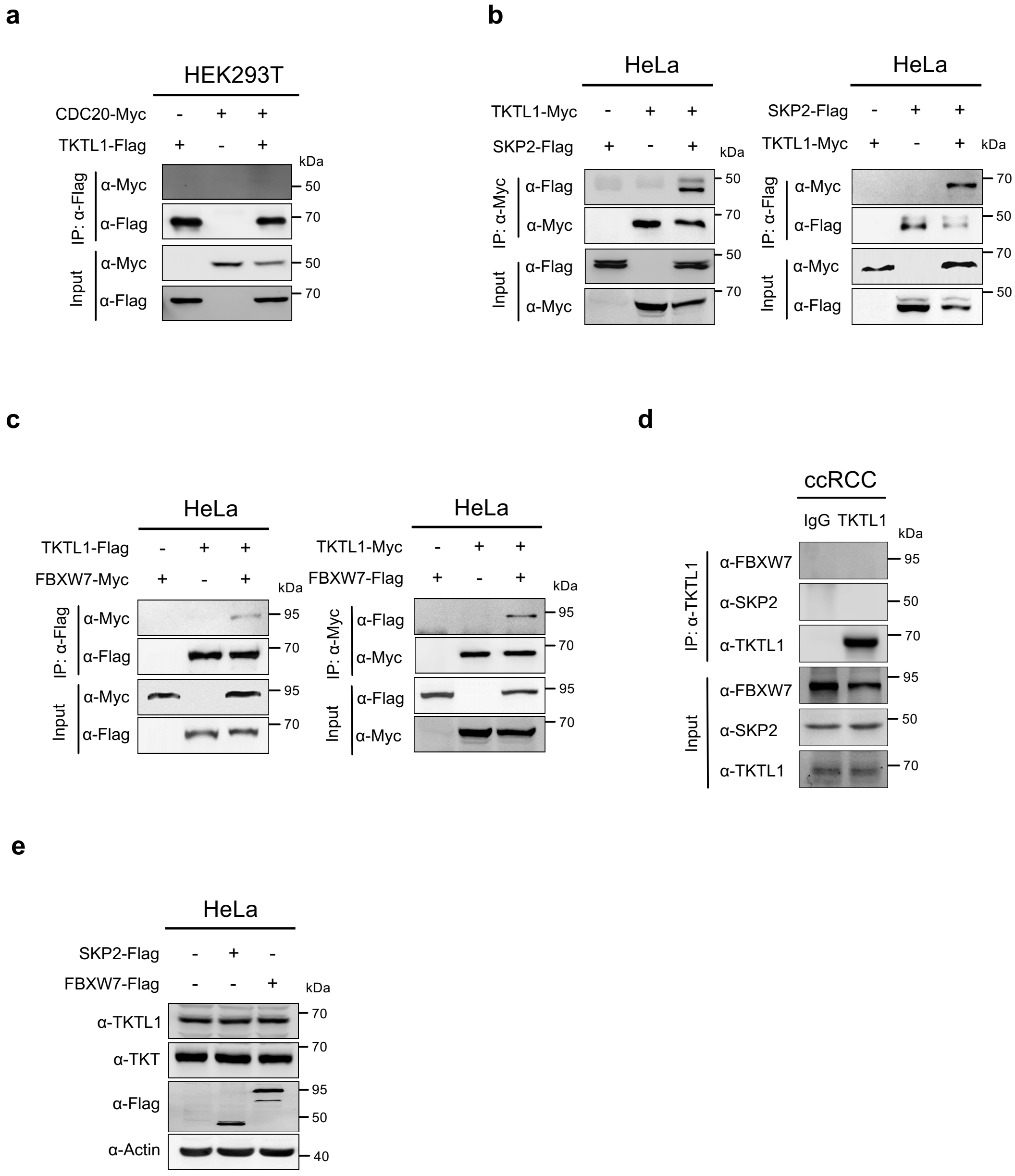

Supplementary Figure 4. Other cell cycle regulating proteins failed to regulate TKTL1 (related to Fig. 2). The interactions between TKTL1 and CDC20 (**a**), SKP2 (**b**), FBWX7 (**c**) were determined by immunoprecipitation. The *in vivo* immunoprecipitation showed TLTL1 did not interact with SKP2 and FBWX7 (**d**). The TKTL1 levels in HeLa and HeLa that overexpressing either SKP2 or FBWX7 were determined by Western blot (**e**).

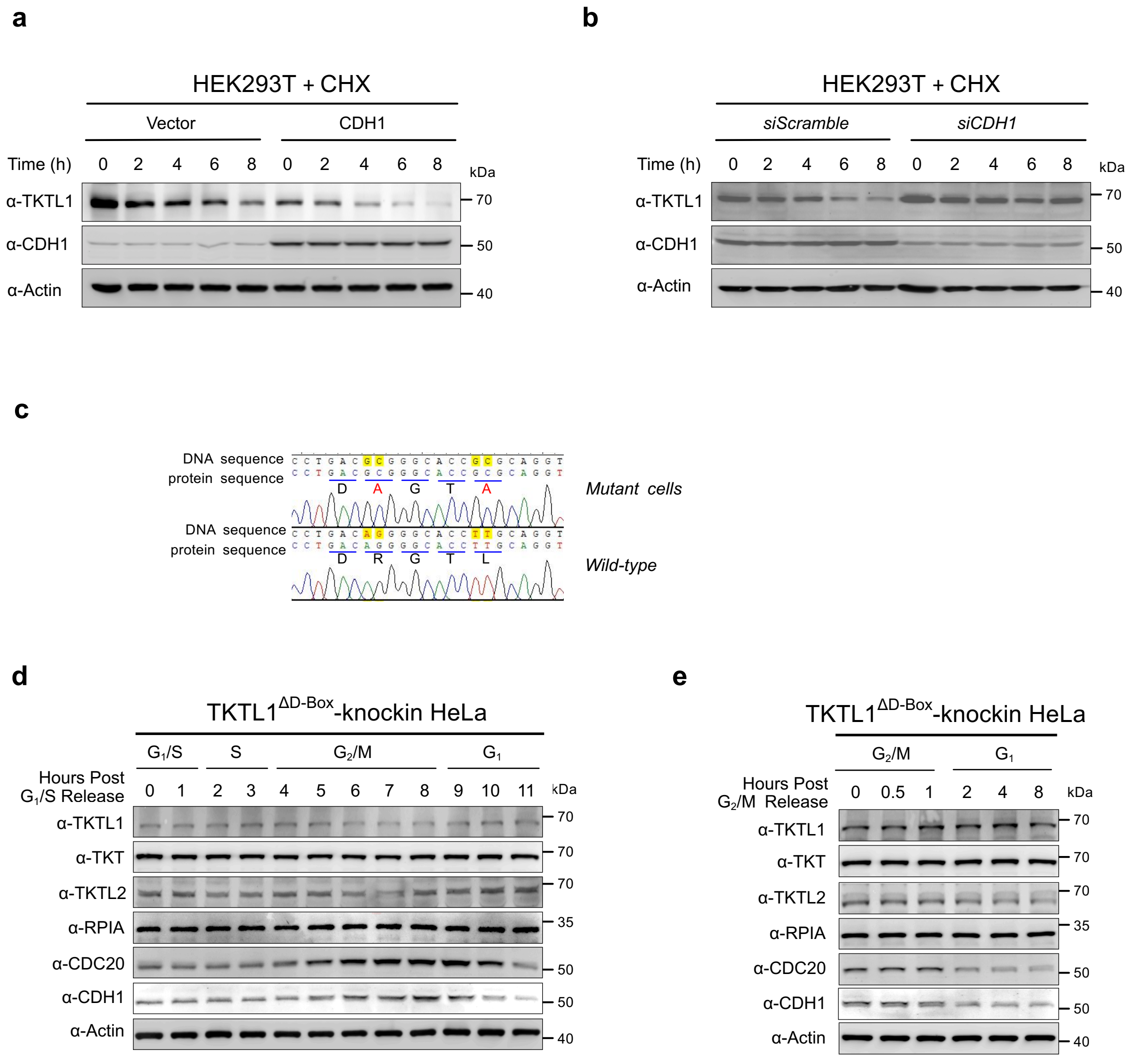

Supplementary Figure 5. CDH1 regulates TKTL1 stability (related to Fig. 2). (**a, b**) CDH1 overexpression promotes the degradation of TKTL1 (**a**), and CDH1 knockdown resulted in increased stability of TKTL1 (**b**). (**c**) TKTL1$^{\Delta\text{D-box}}$-knockin HeLa sequence identifications are shown. (**d, e**) The protein levels of TKTL1, TKT, TKTL2, RPIA, CDC20, and CDH1 were determined at different time points after TKTL1$^{\Delta\text{D-Box}}$-knockin-HeLa cells were released from double thymidine (**d**) and RO3306 (**e**) synchronization.

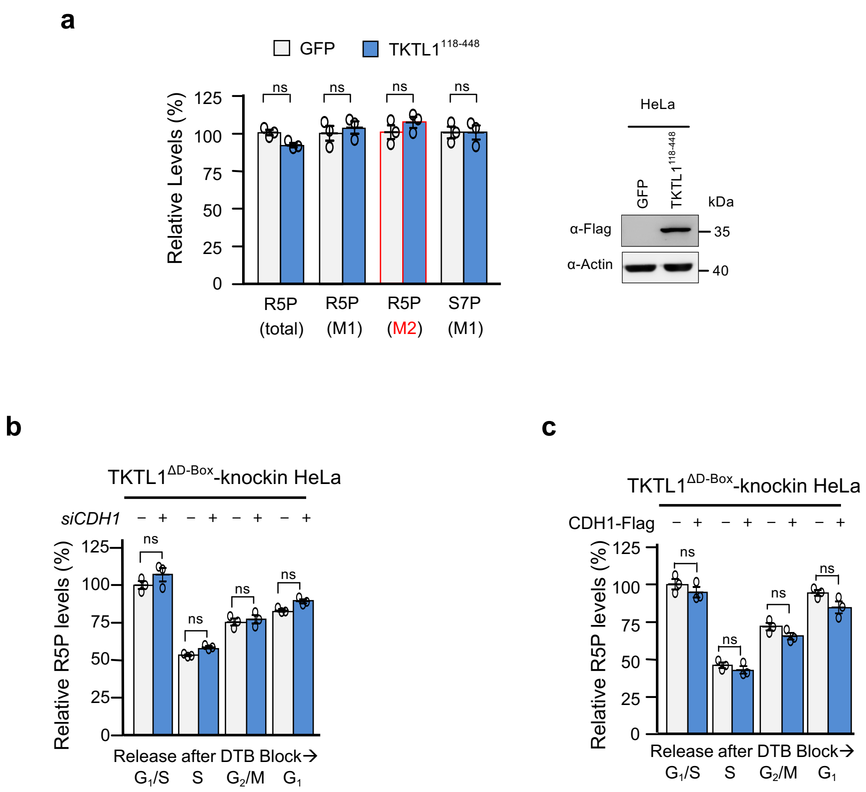

Supplementary Figure 6. TKTL1 mutants didn't reprogram R5P metabolism (related to Fig. 5). (**a**) Relative levels of total, M1 and M2 R5P, and S7P levels were determined in TKTL1$^{118\text{-}448}$ overexpressing HeLa cells and in HeLa cells. Levels of HeLa cells were set as 100% and data are presented by means ± SEM of 3 independent experiments, Student's *t* test, ns not significant. (**b, c**) The R5P levels of TKTL1$^{\Delta D\text{-}Box}$-knockin HeLa cells were compared with that in CDH1 knocked down HeLa cells (**b**) and CDH1 overexpressing HeLa cells (**c**). Data are shown by means ± SEM of 3 independent experiments, Student's *t* test, ns not significant.

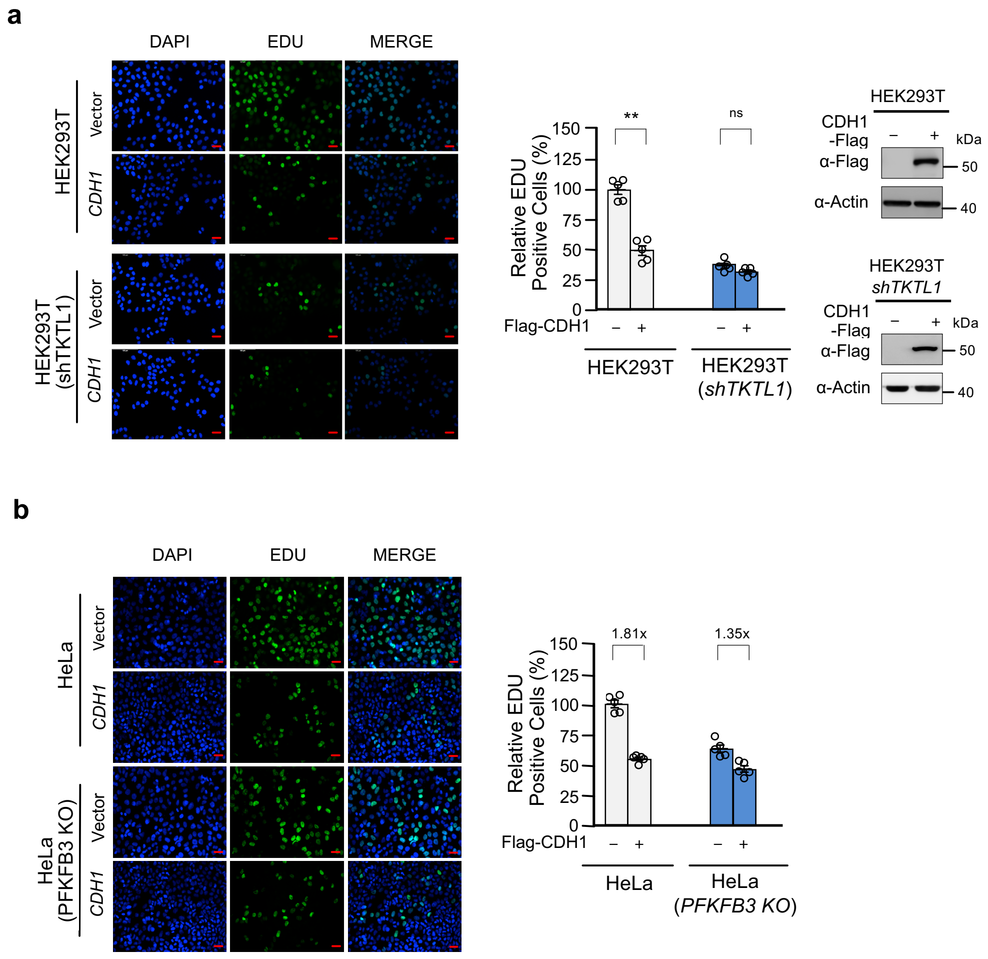

Supplementary Figure 7. APC/C^{CDH1} regulates DNA synthesis through TKTL1 (related to Fig. 7). The CDH1 overexpression effects on DNA synthesis were detected by EDU staining (left) in HEK293T and TKTL1 knockdown HEK293T cells (**a**), and HeLa and PFKFB3 knockout HeLa cells. Data were presented as mean ± SEM, Student's *t* test, **p < 0.01, ns not significant. (**b**). Bar scale: 100 μm. Five biological independent experiments were used to present the data of the relative EDU staining positive cells (right, to that of control HEK293T cells). Data were presented as mean ± SEM.

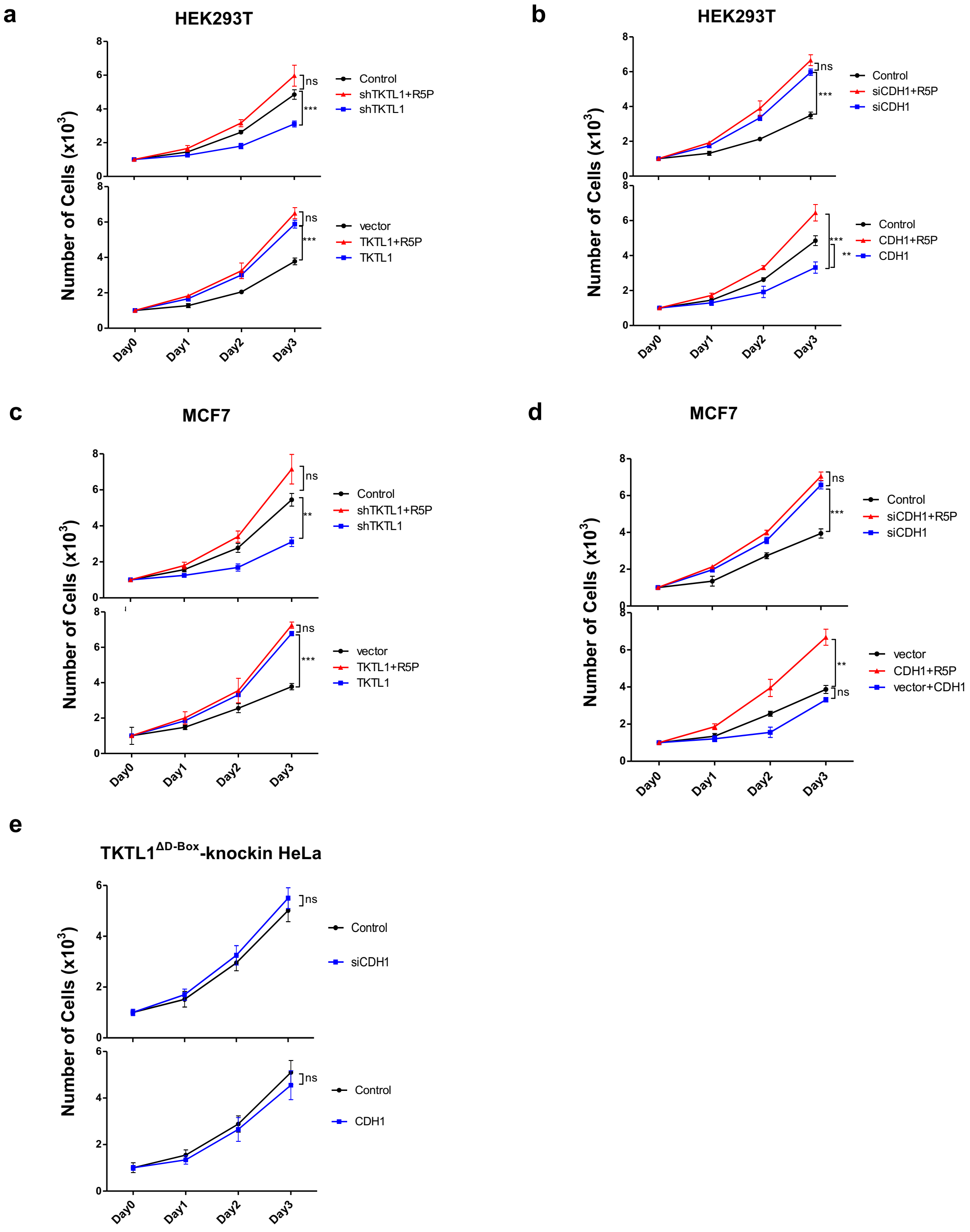

Supplementary Figure 8. APC/C^{CDH1} regulates cell growth through regulating TKTL1 and R5P levels (related to Fig. 7). The growth of HEK293T (**a, b**) and MCF cells (**c, d**) was determined (n=6 biologically independent samples) under TKTL1 overexpression and knockdown (**a, c**), CDH1 overexpression and knockdown (**b, d**), and these conditions with 10 mM R5P supplementation in the culture media (**a-d**). (**e**) The growth of TKTL1$^{\Delta D\text{-}Box}$-knock-in cells was determined under CDH1 overexpression and knockdown.

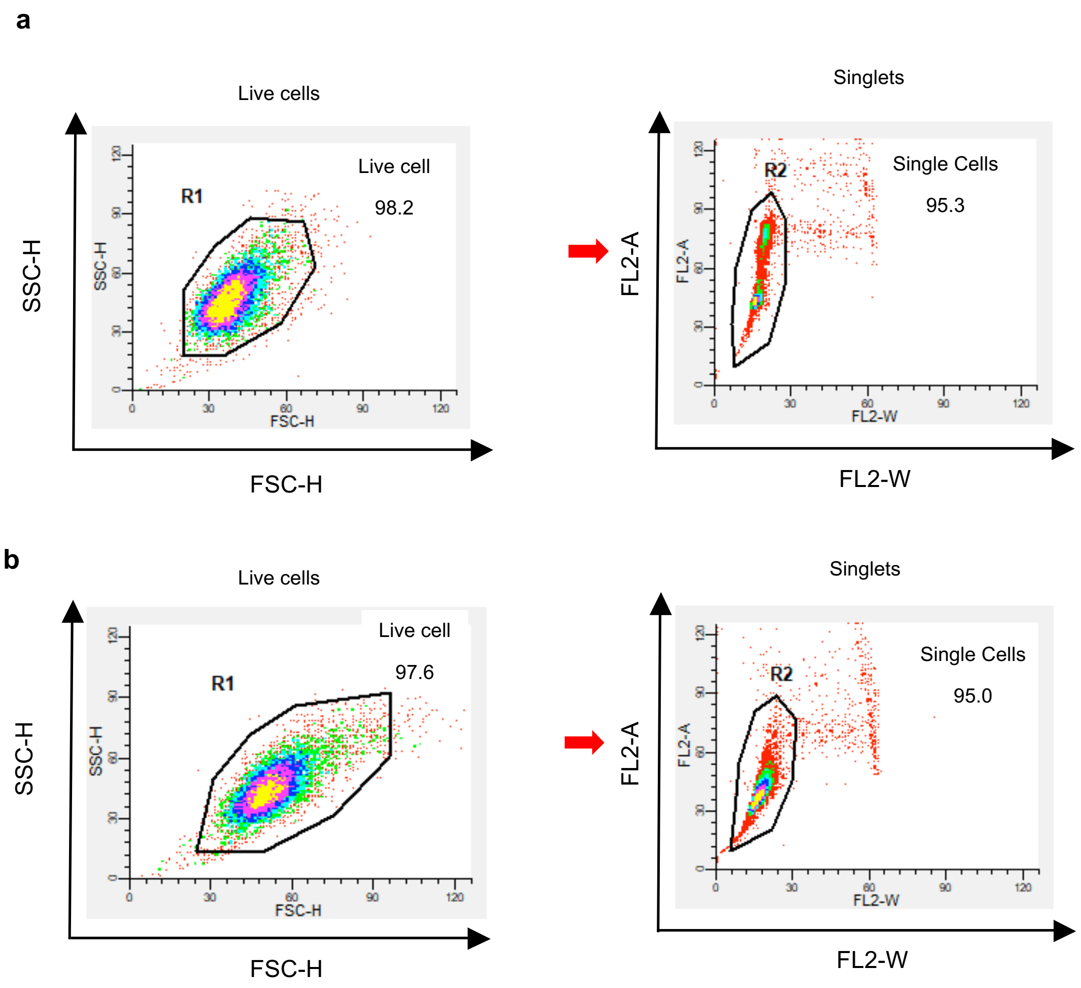

Supplementary Figure 9. Gating strategy of flow cytometry for normal HeLa cells **(a)** and DTB Block Released 0hr HeLa cells **(b).** The flow cytometry results were shown in Supplementary Figure 1.

Supplementary Figure 10. Uncropped and unprocessed scans of gels and blots.

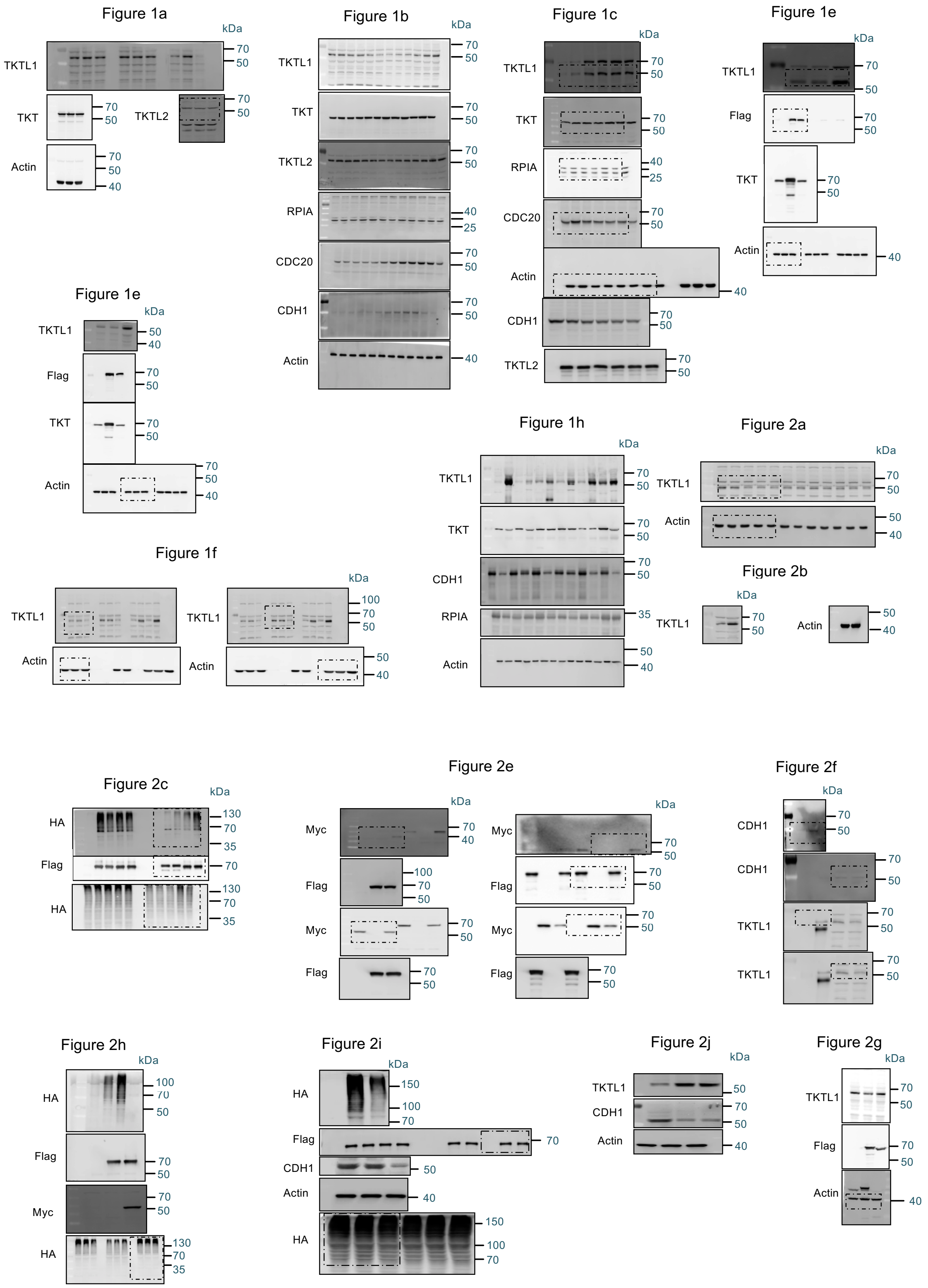

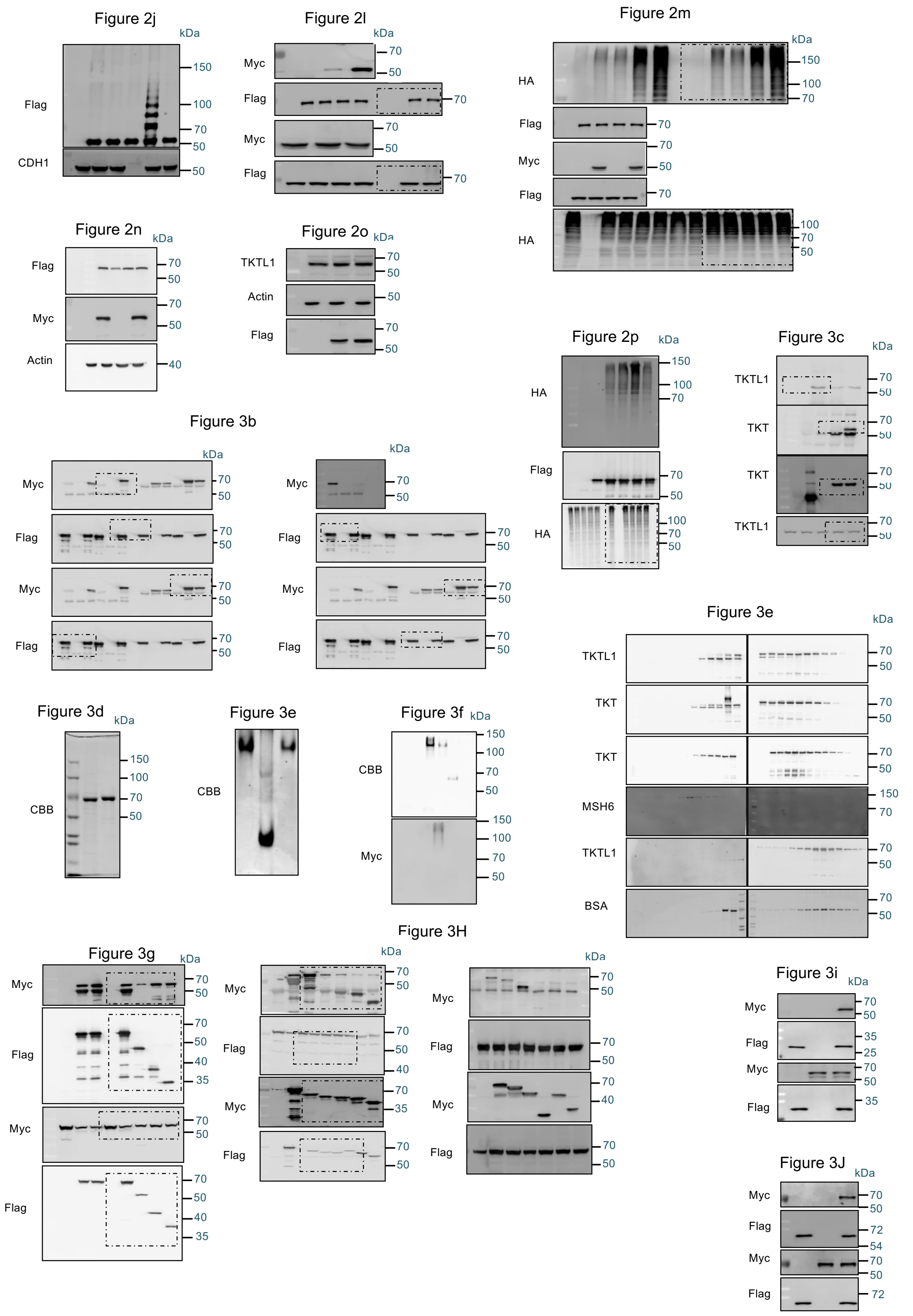

Figure 2j
kDa
Flag
150
100
70
50
CDH1
50
Figure 2l
kDa
Myc
70
50
Flag
70
Myc
70
50
Flag
70
Figure 2m
kDa
HA
150
100
70
Flag
70
Myc
70
50
Flag
70
HA
100
70
50
Figure 2n
kDa
Flag
70
50
Myc
70
50
Actin
40
Figure 2o
kDa
TKTL1
70
50
Actin
50
Flag
70
50
Figure 2p
kDa
HA
150
100
70
Flag
70
50
HA
100
70
50
Figure 3c
kDa
TKTL1
70
50
TKT
70
50
TKT
70
50
TKTL1
70
50
Figure 3b
kDa
Myc
70
50
Flag
70
50
Myc
70
50
Flag
70
50
Figure 3e
kDa
TKTL1
70
50
TKT
70
50
TKT
70
50
MSH6
150
70
TKTL1
70
50
BSA
70
50
Figure 3d
kDa
CBB
150
100
70
50
Figure 3e
CBB
Figure 3f
kDa
CBB
150
100
70
50
Myc
150
100
70
50
Figure 3H
kDa
Myc
70
50
Flag
70
50
40
Myc
70
35
Flag
70
50
Myc
70
50
Flag
70
50
Myc
70
40
Flag
70
50
Figure 3g
kDa
Myc
70
50
Flag
70
50
40
35
Myc
70
50
Flag
70
50
40
35
Figure 3i
kDa
Myc
70
50
Flag
35
25
Myc
70
50
Flag
35
Figure 3J
kDa
Myc
70
50
Flag
72
54
Myc
70
50
Flag
72

Figure 5a

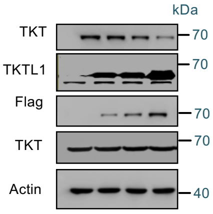

Figure 5b

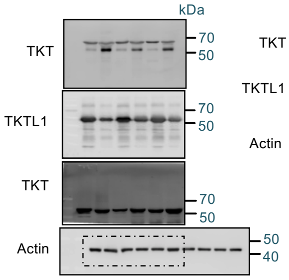

Figure 5c

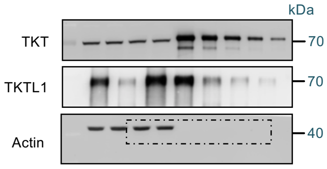

Figure 5d

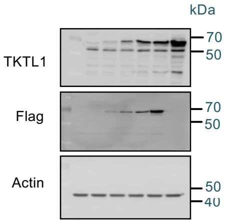

Figure 5e

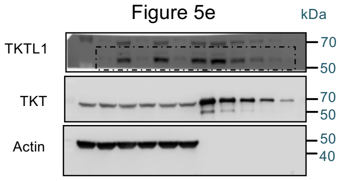

Figure 5g

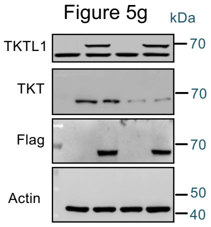

Figure 5j

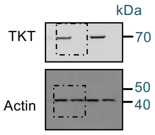

Figure 5k

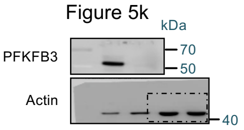

Figure 6a

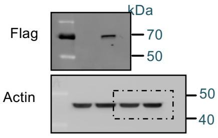

Figure 6b

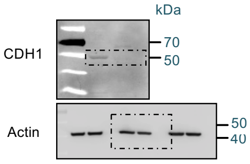

Figure 6d

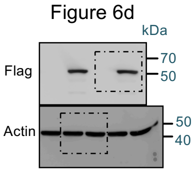

Figure 6e

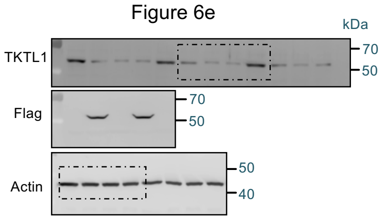

Figure 6f

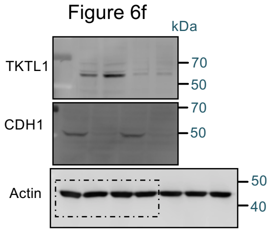

Figure 6i

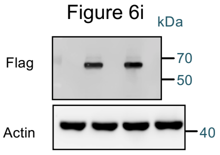

Figure 6g

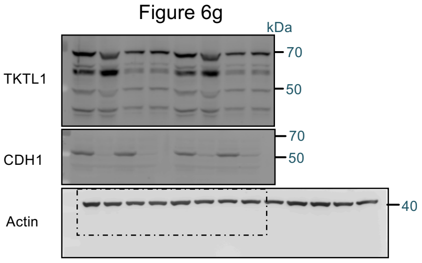

Figure 6h

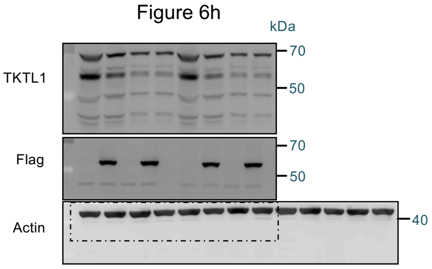

Figure 7a

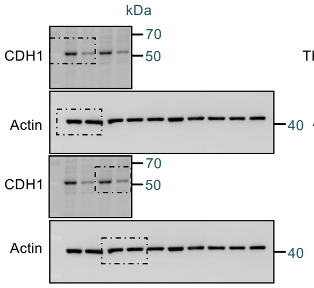

Figure 7b

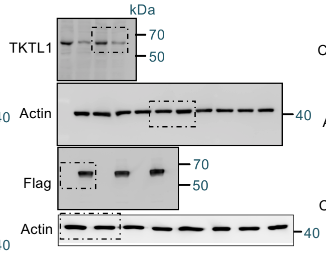

Figure 7c

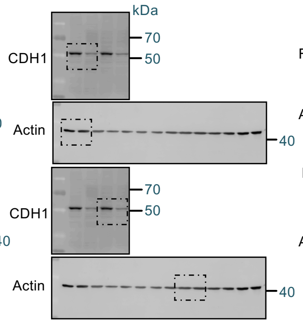

Figure 7d

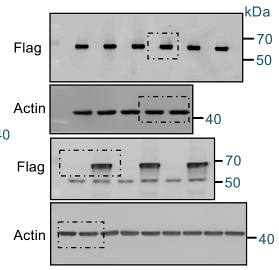

Figure 7e

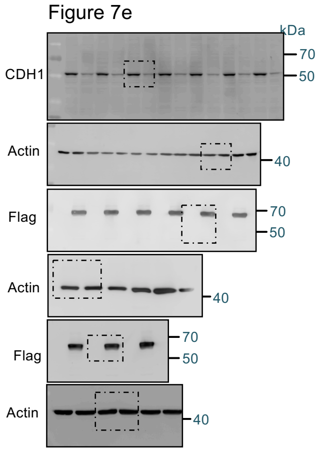

Figure 7f

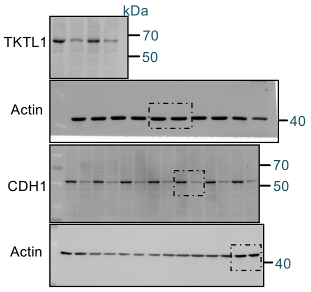

Figure 7g

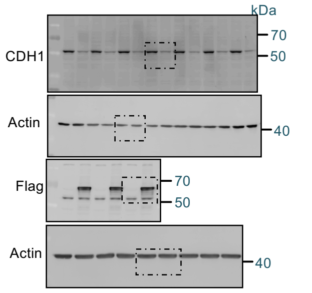

Suppl Fig2

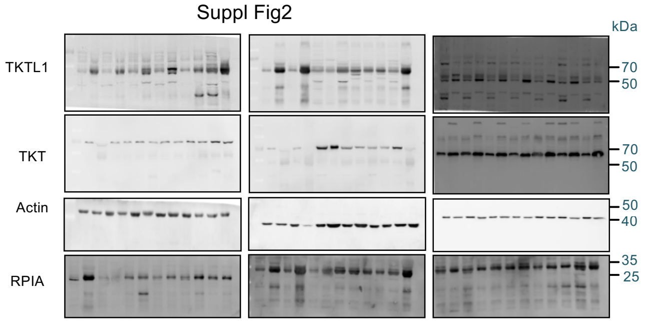

Suppl Fig 4a

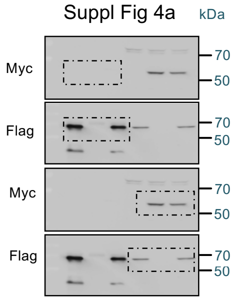

Suppl Fig 4b

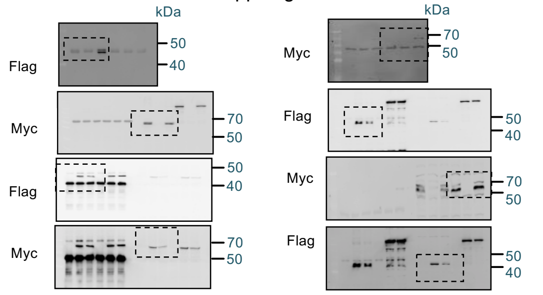

Suppl Fig 4c

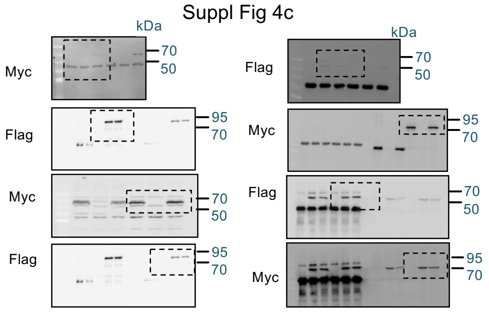

Suppl Fig 4d

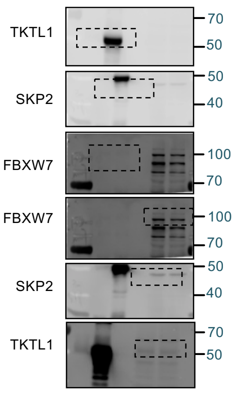

Suppl Fig 4e

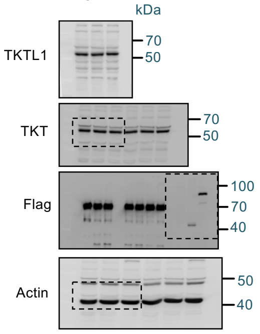

Suppl Fig 5a

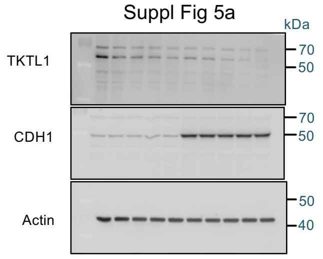

Suppl Fig 5b

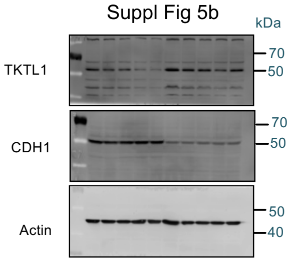

Suppl Fig 5d

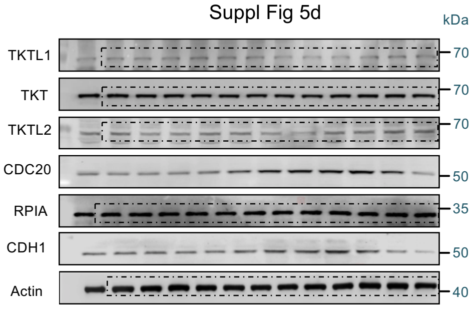

Suppl Fig 5e

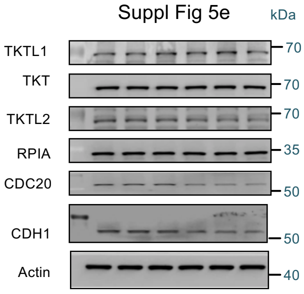

Supplementary Table 1

**Polymerase chain reaction (PCR) Primers**	
TKT-Flag forward:	GCGTCGACATGGAGAGCTACCACAAGCCTGACC
TKT-Flag reverse:	CGGAATTCCTAGGCCTTGGTGATGAGGCCCCTC
TKT-Myc forward:	CCCTCGAGATGGAGAGCTACCACAAGCCTGACC
TKT-Myc reverse:	CGGAATTCCTAGGCCTTGGTGATGAGGCCCCTC
TKTL1-Flag forward:	CCCAAGCTTATGGCGGATGCTGAGGCGAGG
TKTL1-Flag reverse:	CGGAATTCTTAGTTCAGCAACATGCATTTCACGGC
TKTL1-Myc forward:	CGGAATTCATGGCGGATGCTGAGGCGAGG
TKTL1-Myc reverse:	CCCAAGCTTCCTTAGTTCAGCAACATGCATTTCACGGC
CDH1-Flag forward:	CCCAAGCTTATGGAGATTTTGATCACGGTAACCG
CDH1-Flag reverse:	CGGGATCCCTAGTCGTCCTCGCCGCCTCCGTAC
CDH1-Myc forward:	CTAGCTAGCATGGACCAGGACTATGAGCGGC
CDH1-Myc reverse:	CGGAATTCCCGGATCCTGGTGAAGAGGTTGAGC
βTrCP-Flag forward:	CCGCTCGAGATGGACCCGGCCGAGGCGGTGCTGCAAG
βTrCP-Flag reverse:	CGGGATCCGTTCTGGAGATGTAGGTGTATGTTCGAG
SKP2-Flag forward:	GATGACAAGAAGCTTATGCACAGGAAGCACCTC
SKP2-Flag reverse:	CCATCGATTGAATTCTTATTGTTTTAAAACAAG
FBXW7-Flag forward:	GATGACAAGAAGCTTATGAATCAGGAACTGCTC
FBXW7-Flag reverse:	CCATCGATTGAATTCTCACTTCATGTCCACATC
**Truncated TKT and TKTL1 Primers**	
TKTL1-1-448 reverse	CGGAATTCTTATCGGGTGGTCCGAATGAAGCACATC
TKTL1-1-292 reverse	CGGAATTCTTACCGAGTAGCTATCTTGTCACC
TKTL1-1-218 reverse	CGGAATTCTTACTTGAAGGTCTTGGCCACCACAGCA
TKTL1-84-596 forward	CCCAAGCTTAGACTGTCGTTTGTGGATGTGGCAAC
TKTL1-118-596 forward	CCCAAGCTTTACCGGGTGTTCTGCCTCATGAGTG
TKTL1-146-596 forward	CCCAAGCTTCTGGACAATCTTGTGGCAATCTTTG
TKTL1-205-596 forward	CCCAAGCTTGTGAAGCACAAGCCCACTGCTGTGG
TKTL1-235-596 forward	CCCAAGCTTCCAATGCCGAGAGAAAGAGCAGATG
TKTL1-118-448 forward	CCCAAGCTTTACCGGGTGTTCTGCCTCATGAGTG
TKTL1-118-448 reverse	CGGAATTCTTATCGGGTGGTCCGAATGAAGCACATC
TKTL1$^{\triangle 119\text{-}447}$ forward	AGTTTCTGGGCTGGCCCTGTCGAAGTACTT
TKTL1$^{\triangle 119\text{-}447}$ reverse	AGGGCCAGCCCAGAAACTATGGTTATTTAC

TKTL1$^{\triangle 148\text{-}473}$ forward	ATTTTCTGGGCTGGCCTTGTCGAAGTATTT
TKTL1$^{\triangle 148\text{-}473}$ reverse	AAGGCCAGCCCAGAAAATGCCATCATCTAT
***siRNA* sequence**	
CDH1 siRNA-1:	5'- GCCAGATCGTCATCCAGAA
CDH1 siRNA-2:	5'- CCAACTGGAGCGTGAACTT
***shRNA* sequence**	
shTKTL1:	CATTCATCCCTAGTTCGGAAATTCA
shTKT:	TCGCCAGCATCTATAAGCTGGACAA
**Quantitative RT-PCR Primers**	
TKT forward:	5'- GTGGAAAAAGAGGACAGCCA
TKT reverse:	5'- ACAAGCCTGACCAGCAGAAG
TKTL1 forward:	5'- GTCGTTGTCCGGATTCTCTG
TKTL1 reverse:	5'- GAATCCATTCCATCAGGGC
β−actin forward:	5'-GCCGACAGGATGCAGAAGGAGATC A
β−actin reverse:	5'- AAGCATTTGCGGTGGACGATGGA

Supplementary Table 1. Sequences of primers of siRNA, shRNA, PCR and Q-PCR used in this study are listed.
