# Supplementary material for: APC/CCDH1 synchronizes ribose-5-phosphate levels and DNA synthesis to cell cycle progression
Source: Nat Commun. 2019 Jun 7;10:2502. doi: 10.1038/s41467-019-10375-x (PMC6555833; doi:10.1038/s41467-019-10375-x)
Supplement: Supplementary file 3 — Reporting Summary [file 41467_2019_10375_MOESM3_ESM.pdf]

nature research

Corresponding author(s): Shi-Min Zhao

Last updated by author(s): Apr 4, 2019

Reporting Summary

Nature Research wishes to improve the reproducibility of the work that we publish. This form provides structure for consistency and transparency in reporting. For further information on Nature Research policies, see Authors & Referees and the Editorial Policy Checklist.

Statistics

For all statistical analyses, confirm that the following items are present in the figure legend, table legend, main text, or Methods section.

n/a	Confirmed	
☐	☒	The exact sample size (*n*) for each experimental group/condition, given as a discrete number and unit of measurement
☐	☒	A statement on whether measurements were taken from distinct samples or whether the same sample was measured repeatedly
☐	☒	The statistical test(s) used AND whether they are one- or two-sided<br>*Only common tests should be described solely by name; describe more complex techniques in the Methods section.*
☒	☐	A description of all covariates tested
☒	☐	A description of any assumptions or corrections, such as tests of normality and adjustment for multiple comparisons
☐	☒	A full description of the statistical parameters including central tendency (e.g. means) or other basic estimates (e.g. regression coefficient) AND variation (e.g. standard deviation) or associated estimates of uncertainty (e.g. confidence intervals)
☒	☐	For null hypothesis testing, the test statistic (e.g. *F*, *t*, *r*) with confidence intervals, effect sizes, degrees of freedom and *P* value noted<br>*Give P values as exact values whenever suitable.*
☒	☐	For Bayesian analysis, information on the choice of priors and Markov chain Monte Carlo settings
☒	☐	For hierarchical and complex designs, identification of the appropriate level for tests and full reporting of outcomes
☒	☐	Estimates of effect sizes (e.g. Cohen's *d*, Pearson's *r*), indicating how they were calculated

Our web collection on statistics for biologists contains articles on many of the points above.

Software and code

Policy information about availability of computer code

Data collection	No code was used.
Data analysis	No software was used.

For manuscripts utilizing custom algorithms or software that are central to the research but not yet described in published literature, software must be made available to editors/reviewers. We strongly encourage code deposition in a community repository (e.g. GitHub). See the Nature Research guidelines for submitting code & software for further information.

Data

Policy information about availability of data

All manuscripts must include a data availability statement. This statement should provide the following information, where applicable:

- Accession codes, unique identifiers, or web links for publicly available datasets
- A list of figures that have associated raw data
- A description of any restrictions on data availability

All data and genetic material used in this paper are available from the authors on request. The mass spectrometry proteomics data have been deposited to the ProteomeXchange Consortium via the PRIDE partner repository with the dataset identifier PXD013309.

Field-specific reporting

Please select the one below that is the best fit for your research. If you are not sure, read the appropriate sections before making your selection.

☒ Life sciences ☐ Behavioural & social sciences ☐ Ecological, evolutionary & environmental sciences

Life sciences study design

All studies must disclose on these points even when the disclosure is negative.

Sample size	We used 24 pairs of tumors and adjacent normal tissues from patients with clear cell renal cell carcinoma (ccRCC) in western blotting, immunostaining, and metabolites quantification.
Data exclusions	We compared the protein and metabolites levels between tumors and adjacent normal tissues. No data was excluded.
Replication	We have 3 replications for each experiment. The results are repeatable.
Randomization	Each pair of tumor and adjacent normal tissue are from the same patient.
Blinding	All the samples were collected from ccRCC patients. So this study did not refer to blinding in samples collection.

Reporting for specific materials, systems and methods

We require information from authors about some types of materials, experimental systems and methods used in many studies. Here, indicate whether each material, system or method listed is relevant to your study. If you are not sure if a list item applies to your research, read the appropriate section before selecting a response.

Materials & experimental systems

n/a	Involved in the study	
☐	☒	Antibodies
☐	☒	Eukaryotic cell lines
☒	☐	Palaeontology
☒	☐	Animals and other organisms
☒	☐	Human research participants
☐	☒	Clinical data

Methods

n/a	Involved in the study	
☒	☐	ChIP-seq
☐	☒	Flow cytometry
☒	☐	MRI-based neuroimaging

Antibodies

Antibodies used	TKTL1 antibody (#NBP1-31674, Novus Biologicals). CDC20 antibody (#4823, Cell Signaling Technology). SKP2 antibody (#4358, Cell Signaling Technology). CDH1 antibody (#CC43, Millipore). TKT antibody (#sc-67120, Santa Cruz Biotechnology). RPIA antibody (#181235, Abcam). [illegible]-Actin antibody (A00702, GeneScript). Flag antibody (#M20008, Abmart). Myc antibody (#M20003, Abmart). HA antibody (#M20002, Abmart).
Validation	TKTL1 antibody (manufacture's website: https://www.novusbio.com/products/tktl1-antibody_nbp1-31674. Also see the Figure 1F in the main text.)<br>CDC20 antibody (citation: MGMT inhibition in ER positive breast cancer leads to CDC2, TOP2A, AURKB, CDC20, KIF20A, Cyclin A2, Cyclin B2, Cyclin D1, ERα and Survivin inhibition and enhances response to temozolomide.).<br>SKP2 antibody (citation: The Interaction of miR-378i-Skp2 Regulates Cell Senescence in Diabetic Nephropathy.)<br>CDH1 antibody (citation: PTEN mediates Notch-dependent stalk cell arrest in angiogenesis)<br>TKT antibody (citation: Proteomic Identification of Novel Plasma Biomarkers and Pathobiologic Pathways in Alcoholic Acute Pancreatitis.)<br>RPIA antibody (citation: Systems analysis of intracellular pH vulnerabilities for cancer therapy.)<br>beta-Actin (citation: RanBP9 Overexpression Down-Regulates Phospho-Cofilin, Causes Early Synaptic Deficits and Impaired Learning, and Accelerates Accumulation of Amyloid Plaques in the Mouse Brain.)<br>Flag antibody (citation: JNK1 negatively controls antifungal innate immunity by suppressing CD23 expression.)<br>Myc antibody (citation: Use of peptide tagging to detect proteins expressed from cloned genes: deletion mapping functional domains of Drosophila hsp 70.)<br>HA antibody (citation: A short polypeptide marker sequence useful for recombinant protein identification and purification)

Eukaryotic cell lines

Policy information about cell lines

Cell line source(s)	HEK293T cells (ATCC Number: CRL-11268), HeLa cells (ATCC Number: CCL-2) and MCF7 cells (ATCC Number: HTB-22) were used in this study and they were all purchased from Shanghai Cell Bank.
Authentication	HeLa cell line was authenticated using Short Tandem Repeat (STR) analysis by Shanghai Biowing Applied Biotechnology Company.

Mycoplasma contamination	All cell lines tested negative for mycoplasma contamination.
Commonly misidentified lines (See ICLAC register)	HeLa

Clinical data

Policy information about clinical studies

All manuscripts should comply with the ICMJE guidelines for publication of clinical research and a completed CONSORT checklist must be included with all submissions.

Clinical trial registration	Clinical tumor tissue samples were used in this study. The study protocol was reviewed and approved by the ethics committee of the Affiliated Cancer Hospital of Fudan University.
Study protocol	All the clinical samples were used in western blotting, immunostaining, and metabolites quantification.
Data collection	The data collection was performed in our laboratory during December 2015 and December 2016.
Outcomes	n/a

Flow Cytometry

Plots

Confirm that:

☒ The axis labels state the marker and fluorochrome used (e.g. CD4-FITC).

☒ The axis scales are clearly visible. Include numbers along axes only for bottom left plot of group (a 'group' is an analysis of identical markers).

☒ All plots are contour plots with outliers or pseudocolor plots.

☒ A numerical value for number of cells or percentage (with statistics) is provided.

Methodology

Sample preparation	Approximately 100,000 treated cells were suspended in cold 70% ethanol for 3 h, and incubated for 1 h at 37 °C in PBS with DNase-free RNase A (100 mg/mL) and propidium iodide (50 mg/mL).
Instrument	The BD FACSCalibur™ platform
Software	FlowJo 7.6.1
Cell population abundance	First gate on the single cell population using pulse width vs. pulse area. Then apply this gate to the scatter plot and gate out obvious debris. Commonly 10,000 cells were used for analysis after sorting.
Gating strategy	Gating strategy is graphically shown in Supplementary Information.

☒ Tick this box to confirm that a figure exemplifying the gating strategy is provided in the Supplementary Information.
